# High Transient-Thermal-Shock Resistant Nanochannel Tungsten Films

**DOI:** 10.3390/nano11102663

**Published:** 2021-10-11

**Authors:** Tao Cheng, Wenjing Qin, Youyun Lian, Xiang Liu, Jun Tang, Guangxu Cai, Shijian Zhang, Xiaoyun Le, Changzhong Jiang, Feng Ren

**Affiliations:** 1 School of Physics and Technology, Center for Ion Beam Application, Hubei Nuclear Solid Physics Key Laboratory and MOE Key Laboratory of Artificial Micro- and Nano-Structures, Wuhan University, Wuhan 430072, China; tcheng@whu.edu.cn (T.C.); qwj@hunnu.edu.cn (W.Q.); juntang@whu.edu.cn (J.T.); jyk@whu.edu.cn (G.C.); czjiang@whu.edu.cn (C.J.); 2 School of Physics and Electronics, Key Laboratory of Low Dimensional Quantum Structures and Quantum Control, Hunan Normal University, Changsha 410081, China; 3 Fusion Reactor Design & Material Division, Southwestern Institute of Physics, Chengdu 610041, China; xliu@swip.ac.cn; 4 School of Physics, Beihang University, Beijing 100191, China; zhangsj@buaa.edu.cn

**Keywords:** nanochannel W films, ELMs-like, cracking, GIXRD, residual stress

## Abstract

Developing high-performance tungsten plasma-facing materials for fusion reactors is an urgent task. In this paper, novel nanochannel structural W films prepared by magnetron sputtering deposition were irradiated using a high-power pulsed electron beam or ion beam to study their edge-localized modes, such as transient thermal shock resistance. Under electron beam irradiation, a 1 μm thick nanochannel W film with 150 watt power showed a higher absorbed power density related cracking threshold (0.28–0.43 GW/m^2^) than the commercial bulk W (0.16–0.28 GW/m^2^) at room temperature. With ion beam irradiation with an energy density of 1 J/cm^2^ for different pulses, the bulk W displayed many large cracks with the increase of pulse number, while only micro-crack networks with a width of tens of nanometers were found in the nanochannel W film. For the mechanism of the high resistance of nanochannel W films to transient thermal shock, a residual stress analysis was made by Grazing-incidence X-ray diffraction (GIXRD), and the results showed that the irradiated nanochannel W films had a much lower stress than that of the irradiated bulk W, which indicates that the nanochannel structure can release more stress, due to its special nanochannel structure and ability for the annihilation of irradiation induced defects.

## 1. Introduction

Nuclear fusion energy has a great potential to replace traditional energy (coal, oil, etc.) as one of the major energy sources, due to its clean nature. However, the high-performance damage-tolerant materials in the harsh service environment of the reactor have always been a huge challenge. In the International Thermonuclear Experimental Reactor (ITER), besides high H, He, and neutron fluxes, plasma-facing materials (PFMs) also have to withstand a quasi-stationary heat flux load of 10 MW/m^2^ during normal operation, a slow transient heat load of up to 20 MW/m^2^, and transient thermal loads of 1 GW/m^2^ under transient events, such as Type I edge-localized modes (ELMs), plasma disruption, and vertical displacement modes (VDE) [1,2,3,4]. These events have different pulse durations and energy release ranges, which can play a major role in the erosion rate and the PFM lifetime. Type I ELMs, with energy fluxes of 0.5–4 MJ/m^2^ in timescales of 0.3–0.6 ms, are a ubiquitous feature of the H-mode in tokamaks [5]. Large pulsed thermal loads will cause severe damage to PFMs, such as plastic deformations, cracking, melting, and even creep [6,7,8,9,10].

At present, tungsten (W) is considered to be one of the most promising candidate PFMs for the divertor and the first wall in fusion devices, because of its excellent physical and chemical properties, such as a high melting point and thermal conductivity, low sputtering yield and tritium retention, and great strength at elevated temperatures [11,12,13]. To evaluate the performance of W or tungsten-based materials, pulsed electron beams or ion beams are used to simulate ELM transient thermal shock [2,6]. Some studies have shown that the existence of thermal stress was the primary reason for crack formation on tungsten surfaces [14,15,16]. Li et al. [14] reported that cracking occurred when the residual thermal stress was greater than the yield strength during cooling, by simulating transient thermal stress through finite element analysis. Thermal stress is caused by asymmetrical plastic deformation beneath the specimen surface, which is due to the large temperature gradient under transient thermal loads. In addition, it has been reported that crack formation and propagation are related to the microstructure of tungsten-based materials, such as grain shape and texture [17]. Longitudinal, transversal, and recrystallized tungsten samples were prepared and tested by Wirtz et al., which showed that tungsten materials with different structures had differences in thermal shock resistance, and cracks were more likely to occur and expand at grain boundaries. We all know that nanocrystalline materials have better radiation damage resistance than traditional large-size materials, due to the large number of grain boundaries acting as “sinks” to trap defects [18,19]. However, the local surface temperature is higher and the thermal stress is greater due to their worse thermal conductivity [20]. Secondly, they will accumulate more thermal stress at grain boundaries, due to the pinning of dislocations. Therefore, nanocrystalline materials with a large number of grain boundaries are more likely to crack. It is very challenging realize multiple performance requirements in a material at the same time. Therefore, it is necessary to find a unique structure to improve resistance to transient thermal loads, without sacrificing high resistance to radiation damage.

To reduce the generation and propagation of cracks, it is important to reduce the thermal stress accumulation in the material. The accumulation of thermal stress is mainly due to the accumulation of dislocations caused by irregular plastic deformation under the temperature gradient, which is easy to pin to the grain boundary and release on the free surfaces or interfaces, on the contrary [17]. Therefore, it is necessary to introduce a large number of free surfaces or interfaces to release stress in the structural design. In this work, novel nanochannel structure W films with a large number of free surfaces were prepared by ultrahigh vacuum DC magnetron sputtering deposition and their thermal shock resistance was evaluated by pulsed electron or ion beam devices. The nanochannel structure can, not only maintain excellent radiation resistance [21,22], but also provide a large number of nanochannels to release stress. The results show that the nanochannel W films have a higher absorbed power density related cracking threshold than commercial bulk W and have a lower residual stress after irradiation. The present work represents a promising strategy for the design of potential PFMs with excellent performance.

## 2. Experiments

### 2.1. Material Preparation

The nanochannel W films were deposited on commercial W substrates at 600 °C using ultrahigh vacuum DC magnetron sputtering deposition (ULVAC, ACS-4000-C4, made by ULVAC. Inc., Kanagawa, Japan). Nanochannels were generated during the deposition process, due to the high gas flow (Argon), which was 20 standard cubic centimeter per minute (sccm). To obtain different nanochannel densities, the sputtering powers were set to 150 and 50 W, respectively. For simplicity, the films with thicknesses of ~1 μm and 10 μm deposited at different sputtering powers were named as W-150W-1, W-50W-1, and W-150W-10, respectively. At the same time, commercial polycrystalline bulk W slices (99.95 wt% purity, produced by ATTL Advanced Materials Co., Ltd. Beijing, China) with dimensions of 10 × 10 × 4 mm^3^ were prepared as substrates and contrast samples. The bulk W slices were mechanically polished into a mirror finish and then cleaned with acetone ultrasonically.

### 2.2. ELMs-like Transient Thermal Load Tests

In order to evaluate the thermal shock resistance of the nanochannel W films, tests were performed with an electron beam device, EMS-60 (60 kW Electron-beam Material-test Scenario, home-made) at Southwestern Institute of Physics, Chengdu, China [23]. In this experiment, the nanochannel films W-150W-1, W-50W-1, and bulk W were irradiated with ELM at relevant power densities of 0.16, 0.28, and 0.43 GW/m^2^ at room temperature (RT). The acceleration voltage of the electron beam was set to 120 kV, and the electron beam loaded area was approximately 4 × 4 mm^2^. The electron absorption coefficient of W in EMS-60 was 0.46, which corresponds to the value observed in the absorbed current measurements, and did not take into account the emitted low energy secondary electron and backscattered electron [7]. One hundred cycles were applied at a certain input current with a 1 ms pulse duration, and the inter-pulse time was 5 s, to allow complete cool down using water-cooling in the specimen holder made of CuZrCr.

Compared to electron beam irradiation, a high-intensity pulsed ion beam (HIPIB) has higher power densities (>GW/m^2^) and a shorter duration time (~ns), which can explore the effect of materials at higher heat power density depositions in nuclear fusion reactors from another point of view [24]. It has a higher temperature gradient and thermal stress, causing material failure due to its characteristics of rapid temperature rise (10^8^–10^11^ K/s) and cooling (10^7^–10^9^ K/s) [25]. Electron beam and HIPIB irradiation can be used to evaluate the thermal shock resistance of materials from two perspectives. HIPIB irradiation was performed on the TEMP-4M accelerator in the Tomsk Polytechnic University, home-made, in Tomsk, Russia [26,27]. Here, since the heat penetration depth of W in a pulsed ion beam does not exceed 10 μm [25], the W-150W-10 film was prepared and used as the test sample. The ion beam consisted of protons (15%) and carbon ions (85%), and the ion current density was set to 90–100 A/cm^2^; while the average energy of the beam was 175 keV. The full pulse-width at half maxima was ~80 ns, with a pulse interval of 10 s. The energy density was ~1 J/cm^2^ with cycles of 10, 50, and 100, respectively.

### 2.3. Residual Stress Measurement

**Figure 1:**
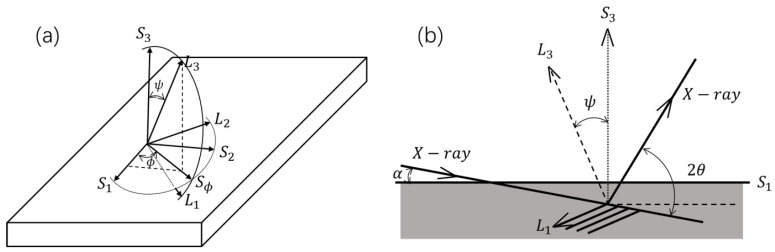
Definition of the laboratory coordinate system $L_{i}$ and sample coordinate system $S_{i}$, and the angles φ and ψ (**a**). The asymmetric diffraction geometry of $g-\sin^{2}\psi$ method (**b**).

X-ray diffraction (XRD) is an effective tool for measuring the residual stress of materials [28,29]. Lattice spacing changes caused by residual stress can be detected by the shift of Bragg angles ($\theta -\theta_{0}$), where $\theta_{0}$ corresponds to the Bragg angle of the stress-free state. The grazing-incidence XRD (GIXRD) method (known as $g-\sin^{2}\psi$ method) [30,31,32] is suitable for analysis of stress in thin films, since it employs an asymmetric diffraction geometry and controls the penetration depth of the X-ray by changing the angle α, which can effectively measure the diffraction peaks of thin films, as shown in Figure 1. The incident angle α is fixed, and diffraction peaks of different {hkl} planes are collected in a single 2θ scan. The ψ angles depend on the {hkl} reflections and the constant incident angle α, i.e.,: $\psi_{\left\{\mathrm{hkl}\right\}}=\theta_{\left\{\mathrm{hkl}\right\}}-\alpha$, where $\theta_{\left\{\mathrm{hkl}\right\}}$ is the Bragg angle for the corresponding {hkl} plane. It is assumed that the stress tensor of the film is biaxial and symmetrical, the X-ray residual stress can be calculated using Equation (1) [28]:

(1) $$\varepsilon_{\phi }=\frac{d_{\phi }-d_{0}}{d_{0}}=\frac{1+\nu }{E}\sigma_{\phi }\sin^{2}\psi -\frac{\nu }{E}\left(\sigma_{11}+\sigma_{22}\right)$$

Here, $\varepsilon_{\phi }$ is the strain along the $L_{3}$ direction, $\sigma_{\phi }$ is the stress component along the $S_{\phi }$ (in plane) direction, and $\sigma_{11}$ = $\sigma_{22}$, $d_{\phi }$ is the lattice spacing obtained from the position of the diffraction peak; $d_{0}$ is the lattice spacing of the stress-free state, and ν and E are the Poisson ratio and the elastic modulus of the sample, respectively.

To record as many diffraction peaks as possible, a scanning range of 2θ was set from 30° to 110° with an incident angle of α = 5°. The values of the Poisson ratio and the elastic modulus used in the present study were ν = 0.28 and E = 410 GPa [11], respectively. Reliable stress values were obtained by a series of data processes, including smoothing, subtracting the background, stripping Kα_2_ diffraction, fitting the “Pseudo Voigt” profile for diffraction peak shape, and determining diffraction peak position in the center of full width at half maxima (FWHM). XRD tests were performed using the SmartLab 3KW at Wuhan University made by Rigaku in Tokyo, Japan.

### 2.4. Characterization Methods

The surface and cross-sectional morphologies of the irradiated samples were characterized by scanning electron microscope (SEM, S-4800 made by Hitachi, Ltd. in Tokyo, Japan) and FIB (Versa 3D made by FEI in Hillsboro, OR, USA). Cross-sectional transmission electron microscopy (XTEM) images were obtained using a JEOL JEM-2100 (TEM) operating at 200 kV made by JEOL Ltd. in Tokyo, Japan.

## 3. Results and Discussion

### 3.1. Microstructure of Nanochannel W Films

**Figure 2:**
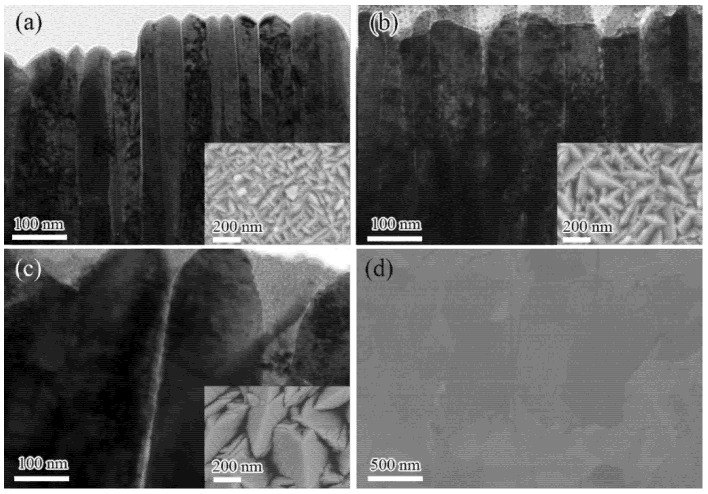
The XTEM image of the W-150W-1 (**a**), the W-50W-1 (**b**), the W-150W-10 (**c**) film, and the surface SEM image of the commercial bulk W (**d**); insets show the corresponding surface SEM images.

The microstructure of the nanochannel W films formed by DC magnetron sputtering deposition were characterized by TEM and SEM. Figure 2a–c shows XTEM images of the three nanochannel W films, while the inserts show the surface SEM image of the corresponding films. It was found that the W films grow in the form of columnar crystals with nanochannels between them. By analyzing the SEM and XTEM images, it was found that the average columnar sizes in the nanochannel W-150W-1, W-50W-1, and W-150W-10 films were approximately 40 ± 10 nm, 80 ± 20 nm, and 200 ± 50 nm (in Figure S1, Supplementary Material), respectively, indicating that the nanochannel W-150W-1 film contained more nanochannels than the nanochannel W-50W-1 film. As the film thickness increases to 10 μm, the size of the crystal column increases. The dark-field XTEM images of the W-150W-1 and W-50W-1 film and the electron diffraction in region A in Figure S2 (Supplementary Material) indicate that the nanocrystalline column in the film is single-crystal, which is beneficial for the transfer of heat, without scattering of the electron at the grain boundaries. It was reported that W with columnar crystals grown by CVD had a higher thermal conductivity than bulk W [23]. Figure 2d shows the polished surface SEM image of commercial bulk W. Figure S2d in the Supplementary Material shows the GIXRD patterns of the three nanochannel W films and commercial bulk W, all of which are stable α phases with body-centered cubic (bcc) structure.

### 3.2. Damage Behavior under Pulsed Electron Beam Bombardment

**Figure 3:**
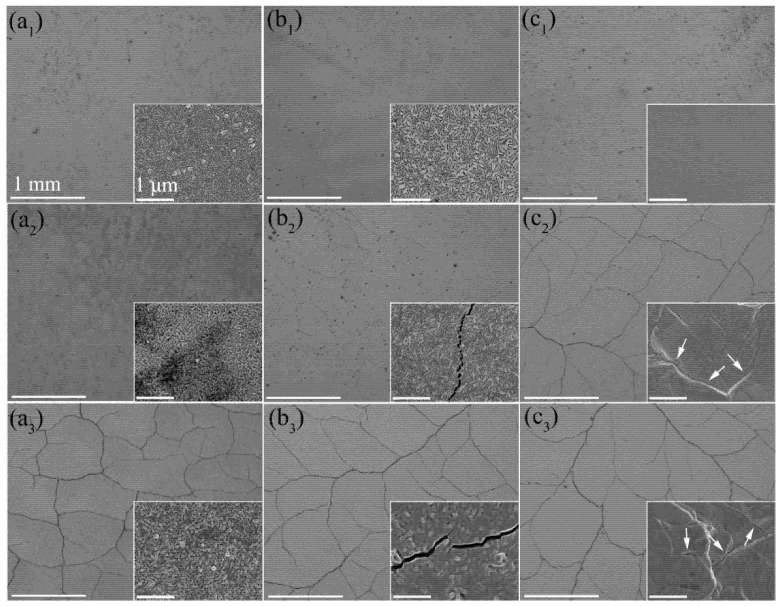
The SEM images of nanochannel W films W-150W-1 (**a_1_**–**a_3_**), W-50W-1 (**b_1_**–**b_3_**), and bulk W (**c_1_**–**c_3_**) exposed to ELM-like transient thermal shock loads with 100 pulses at RT. The absorbed power density was 0.16 GW/m^2^ (**a_1_**,**b_1_**,**c_1_**), 0.28 GW/m^2^ (**a_2_**,**b_2_**,**c_2_**), and 0.43 GW/m^2^ (**a_3_**,**b_3_**,**c_3_**), respectively. Insets show the corresponding magnified images. The white arrows in **c_2_** and **c_3_** indicate the microstructure and microcracks at grain boundaries. The corresponding scale is uniform.

The nanochannel films W-150W-1 and W-50W-1 deposited on bulk W substrates and the bulk W were exposed to pulsed electron beam bombardment with different absorbed power densities (0.16 GW/m^2^, 0.28 GW/m^2^, and 0.43 GW/m^2^) at RT. The overview surface morphologies of these three samples after exposure to ELM-like transient thermal loads are shown in Figure 3.

No cracks were observed on the surface of all three samples when the absorbed power density was 0.16 GW/m^2^.When the absorbed power density increased to 0.28 GW/m^2^, many large cracks were found on the bulk W surface, since the thermal stress exceeded the yield strength at the loaded surface, which is the typical damage under ELM-like transient thermal loads. Moreover, the surface becomes rough, especially at the grain boundaries, since they are the weakest regions in the bulk W [33,34]. Many microcracks with a width of tens of nanometers form on the surface of the nanochannel W-50W-1 film; however, the surface morphology of columnar crystal remains almost unchanged. Notably, no cracks were found on the surface of the nanochannel W-150W-1 film, and the surface morphology also remained unchanged. The accumulation of thermal stress is easy to pin to the grain boundary and, on the contrary, release on the free surfaces or interfaces. The reason for the lack of cracking is that the nanochannel W films with a large number of free surfaces can release stress, so that the internal stress cannot reach its yield strength [35]. With a further increase of absorbed power density to 0.43 GW/m^2^, the surface cracks on the nanochannel W-50W-1 film and bulk W elongate; meanwhile, on the surface of the nanochannel W-150W-1 film, noticeable small cracks start to appear. The surfaces of both the nanochannel W-150W-1 and W-50W-1 films with columnar crystal structures appeared to be slightly melted, while the bulk W suffered significantly higher damage. The above results demonstrate that the nanochannel W films can effectively suppress the formation of cracks and improve the thermal shock resistance.

**Figure 4:**
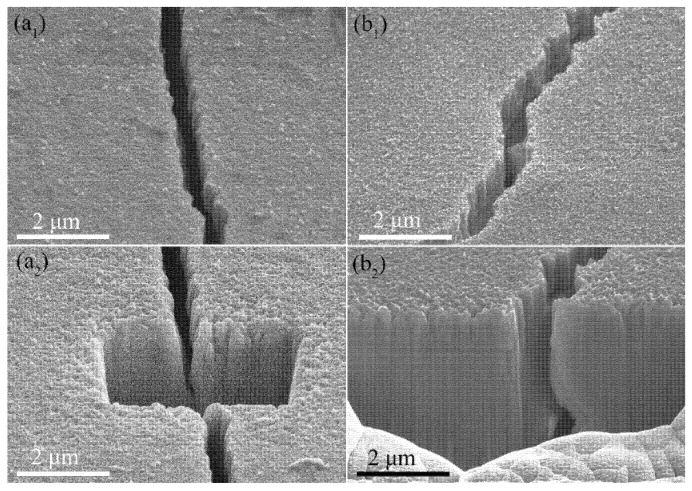
SEM images of the W-150W-1 film (**a_1_**,**a_2_**) at the absorbed power density of 0.43 GW/m^2^, and the W-50W-1 film (**b_1_**,**b_2_**) at 0.28 GW/m^2^.

Since the W-150W-1 film has a larger nanochannel density compared to the W-50W-1 film, it could release more residual stress to delay the cracking of the film when the absorbed power density was 0.28 GW/m^2^. It should be noted that, although the W-150W-1 film has a higher cracking threshold, cracks were observed after irradiation at the absorbed power density of 0.43 GW/m^2^, as shown in Figure 3(a_3_). The characteristic feature of the cracks was very similar to those of the W-50W-1 film and the bulk W. Combined with the results of the SEM images in Figure 4, which show that cracks were formed in the W substrate, the formation of cracks in the film was due to it being torn by the cracks in the W substrate. The penetration depth of a 120 keV electron beam in W is estimated to be approximately 7 µm [36], which is significantly deeper than the total thickness of the nanochannel W films (~1 µm) in this work. Although the W films partially absorb energy from the electron beam, the W substrate also receives thermal shock. Under high power density pulsed electron beam irradiation, the W substrate under the film still cracks, because of its much lower cracking threshold. This phenomenon was also found in the W-50W-1 film, as shown in Figure 4(b_1_,b_2_). Moreover, no cracking was observed in the regions surrounded by large cracks on the surface of nanochannel films, which further indicates that the nanochannel films are more resistant to thermal shocks.

### 3.3. Damage Behavior under HIPIB Bombardment

**Figure 5:**
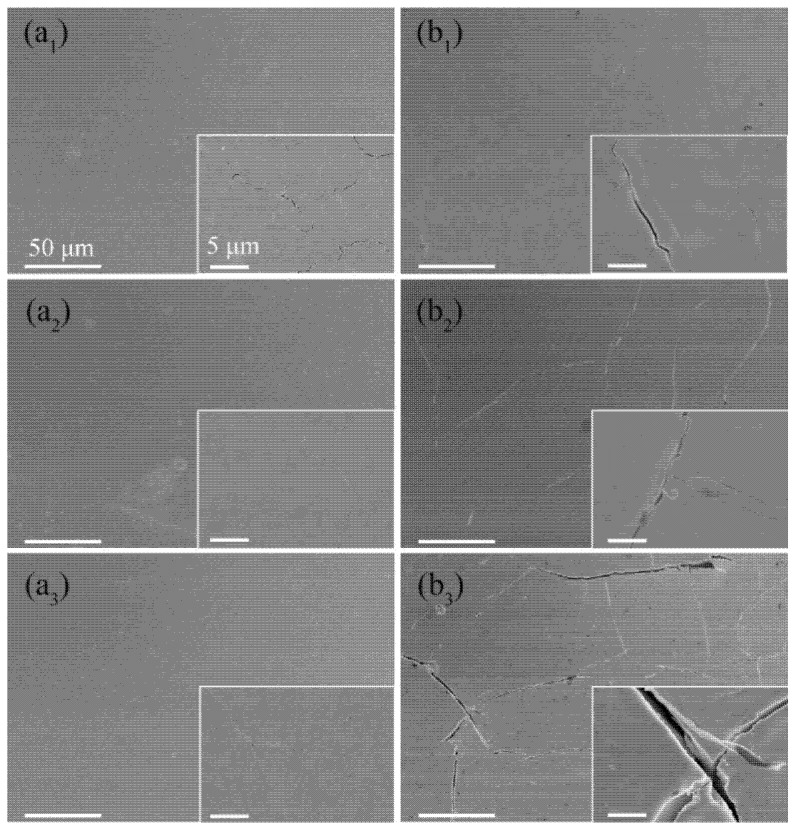
SEM images of the W-150W-10 film (**a_1_**–**a_3_**), and bulk W (**b_1_**–**b_3_**), irradiated by HIPIB at RT; the energy density was ~1 J/cm^2^, with pulses of 10 (**a_1_**,**b_1_**), 50 (**a_2_**,**b_2_**), and 100 (**a_3_**,**b_3_**), respectively. Insets show the corresponding magnified images. The corresponding scale is uniform.

To further evaluate the thermal shock resistance of the nanochannel W films at higher power densities and shorter time scales, HIPIB irradiation was performed. The W-150W-10 film and the bulk W were irradiated with an energy density of 1 J/cm^2^ and a duration of 80 ns. The overview surface morphologies of these samples are shown in Figure 5.

**Figure 6:**
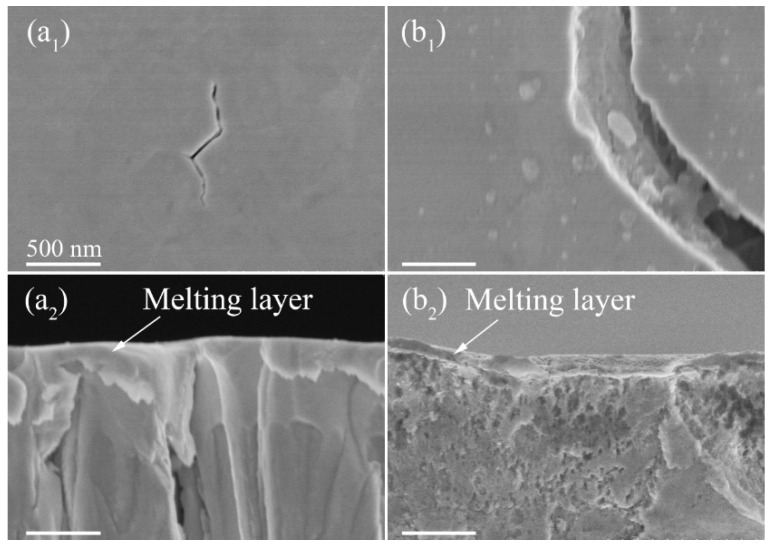
The SEM surface and cross-sectional images of the W-150W-10 film (**a_1_**,**a_2_**) and bulk W (**b_1_**,**b_2_**) irradiated by HIPIB with an energy density of 1 J/cm^2^ and pulses of 10. The corresponding scale is uniform.

It shows that no large cracks were observed in the low magnified SEM of the W-150W-10 film, and only microcracks, with a width of tens of nanometers, appear in the magnified SEM images with the increasing number of irradiation cycles. However, the bulk W showed an obvious cracking phenomenon, which was also observed by Mei et al. at an energy density of 1.4 or 2.0 J/cm^2^ with pulses of 3 [24]. The cracks became more numerous and larger with the increasing number of irradiation cycles. The crack width even reached micron scale as the pulses increased to 100 times. At the same time, the microcracks gradually increased, which was caused by the gradual increase of internal thermal stress. The thickness of the W-150W-10 film decreased from 13.1 to 11.6 μm with the increase in the number of cycles to 100, as shown in Figure S3. Here, we tried to measure this in a similar position. This indicates that HIPIB has a sputtering effect on nanochannel W film. A molten layer of nearly 200 nm on the surface of the W films can be clearly observed in Figure 6(a_2_).

However, beneath the molten layer, the film still maintained a columnar structure, and had the ability to release stress with a further increase of cycle number, while the bulk W could not. Compared to the W-150W-10 film, bulk W suffers more serious damage with an increase of cycles. The cracks that appeared on the surface of the tungsten mean that the accumulated stresses exceeded the ultimate tensile strength of the grain boundaries [24]. It was also found that the surface layer of the bulk W had a molten layer, as shown in Figure 6(b_2_). The high power density and short pulse deposition of HIPIB caused the surface melting of the materials, which was confirmed by the related numerical simulation of the thermal field [37]. According to the above analysis, nanochannel W films show a strong anti-cracking ability, no matter the electron beam or HIPIB irradiation.

### 3.4. Evolutions of Stress under Irradiation

**Figure 7:**
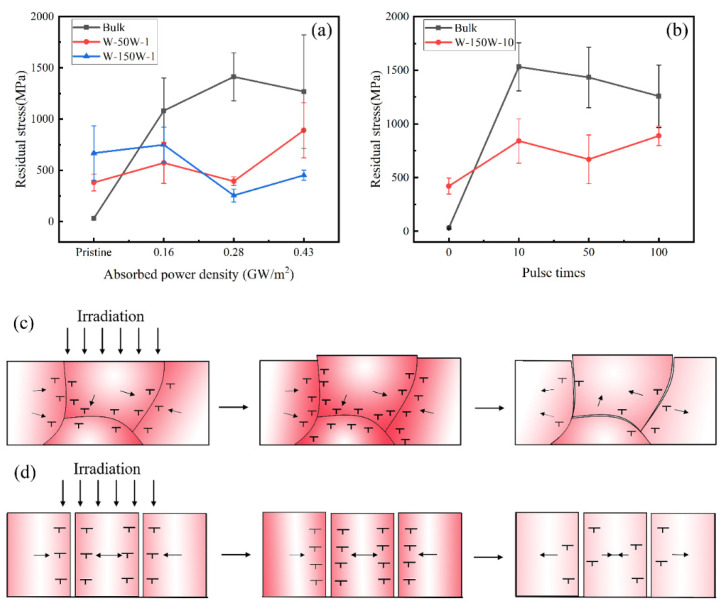
The residual stress of the samples irradiated using a pulsed electron beam (**a**), and HIPIB (**b**). Schematic diagram of the stress evolution of the bulk W (**c**) and the W film (**d**) under irradiation. The sign ┬ represents dislocation; the shades of red represent the stress.

In order to quantify the ability for releasing stress of nanochannel W films and to study the mechanism of the high resistance of nanochannel W films to transient thermal shock, the residual stress of the pristine and irradiated samples is measured, respectively, and the results are shown in Figure 7a,b.

The GIXRD patterns for all the samples are shown in Figure S5, and the residual stress was calculated based on these data. Figure 7a shows the residual stress of the samples irradiated using a pulsed electron beam. The residual stress of the pristine bulk W was near zero, indicating that the residual stress of the substrate was negligible. The original stress of the nanochannel W films was higher than that of the bulk W, because there is tensile stress accumulation during the growth of the thin film by magnetron sputtering deposition at 600 °C [38]. When the absorbed power density reached 0.16 GW/m^2^, the residual stress of the bulk W sharply increased to 1081 MPa, which indicates that a large amount of stress accumulated during this process. However, the stress of the nanochannel W films increased slightly, which shows its outstanding ability to release stress. The residual stress of the bulk W was further increased when the absorbed power density was 0.28 GW/m^2^; the observation of surface cracking proves that the accumulated stress at this absorbed power density was beyond its yield strength [14]. It can be seen in Figure 3(c_2_) that the uneven surface marked by white arrows at the grain boundaries was due to the incomplete recovery of local plastic deformation. However, the residual stress of both nanochannel W films was unexpectedly reduced when the absorbed power density was 0.28 GW/m^2^. When the absorbed power density increased to 0.43 GW/m^2^, the residual stress of the bulk W decreased slightly. The many microcracks observed at the grain boundaries in the corresponding SEM images indicate the release of residual stress [39]. The residual stress of the nanochannel W films increased as the absorbed power density increased to 0.43 GW/m^2^. Although the nanochannel structure can release a large part of the stress, the residual stress is still gradually accumulated in the columns under high power density pulsed electron beam irradiation. Nevertheless, the residual stress (452 MPa) of the W-150W-1 film was much smaller than that of the bulk W (1268 MPa).

The evolution of stress is closely related to the microstructure of the materials. It was revealed that there were different stress evolution modes between the bulk W and the nanochannel W film, due to the great difference in their microstructures. The evolution process of the residual stress of the bulk W under irradiation is presented in Figure 7c, based on the experimental results. In the process of heating, the volume expansion of the material produces compressive stress in the interior, and a large number of dislocations are caused by the non-uniform deformation of the material, due to the huge temperature gradient, which easily pins to the grain boundaries. The shrinkage of volume during the recovery of deformation results in the changing of the internal compressive stress into tensile stress [14]. When the temperature fails to cause the plastic deformation of the material, the deformation recovers completely. When the high temperature causes materials to deform plastically, the surface roughness increases, due to the incomplete recovery of deformation [17]. On the other hand, the dislocations pinned at the grain boundaries cannot be released in time, which leads to the accumulation of stress. When it reaches a certain threshold value, the grain boundaries will crack and release stress. The evolution process of the residual stress of the nanochannel W film is shown in Figure 7d. The nanochannel film is composed of numerous single-crystal columns arranged vertically, with nanometer channels between them. The size of the crystal columns is in the tens of nanometers. During the thermal expansion process under irradiation, each nanocrystalline column has enough space for its thermal expansion, which reduces its internal stress and suppresses its plastic deformation. Furthermore, the dislocations generated internally are more likely to move to the free surface of the nanochannels and be released, making it difficult to accumulate large amounts of stress [35]. Therefore, the recovery of deformation during the cooling process will not lead to excessive tensile stress and cracking.

Regarding the decrease of residual stress in nanochannel W films at the absorbed power density of 0.28 GW/m^2^, this was because the original stress formed during film growth was partially released in the nanochannel film during electron irradiation [40,41]. Moreover, the nanochannel density of the W-50W-1 film was lower than that of the W-150W-1 film. When the absorbed power density was 0.28 and 0.43 GW/m^2^, the stress of the W-50W-1 film was higher than that of the W-150W-1 film. This is because the W-50W-1 film had fewer nanochannels to release the stress. However, the stress of the W-50W-1 film was still much smaller than that of the bulk W.

The X-ray had a penetration depth of nearly 600 nm in W due to the angle of incidence of α = 5°. For the W-150W-10 film irradiated by HIPIB, because of the existence of a stress-free melting layer at the first surface, the measured stress was mainly from the region below the melting layer. As shown in Figure 7b, the residual stress of the bulk W was as high as 1531 MPa under 10 pulses, and the stress value decreased slightly with the increase of pulse number. The formation of surface cracks in the bulk W (Figure 6(b_1_,b_2_)) indicates that the accumulated stress had reached its yield strength under the ion beam bombardment at 10 pulses. Due to the characteristics of HIPIB, with the rapid rising and dropping of temperature on the sample surface, there is a very large temperature gradient at the subsurface under the molten layer, which results in uneven plastic deformation of the grains, leading to the production of a large stress gradient. With the increase of the pulse number to 50 and 100, the expansion of surface crack releases part of the stress. Meanwhile, the thickness of the molten layer increases gradually, as shown in Figure S4 (Supplementary Material), which reduces the effective thickness of the stress layer detected by GIXRD, which may also cause a reduction of measured stress.

The stress change of the W-150W-10 film irradiated by HIPIB (Figure 7b) was similar to that of the W-150W-1 film irradiated by the pulsed electron beam. It was found that the stress value fluctuated below 1000 MPa, indicating the good stress-releasing ability of the nanochannel structure. Meanwhile, although the surface layer was melted, the film under the melting layer still maintained a crystal column structure, which was still an effective way to release the stress.

## 4. Conclusions

In summary, nanochannel W films were prepared by ultrahigh vacuum DC magnetron sputtering deposition and irradiated by high power pulsed electron beam and pulsed ion beam irradiation, to evaluate their performance under ELM-like transient thermal loads. It was found that the thermal shock resistance was closely related to the structure of the materials. For the pulsed electron beam irradiation, the absorbed power density related cracking threshold of the W-150W-1 film was located between 0.28 and 0.43 GW/m^2^, which was significantly higher than that of the bulk W; located between 0.16 and 0.28 GW/m^2^. For the HIPIB irradiation, only microcracks with a width of tens of nanometers were found in the irradiated W-150W-10 film, while large cracks were formed in the irradiated bulk W, where the crack size and number increased with increasing pulse number. Both irradiations proved that the nanochannel W films have better resistance to cracking than the bulk W.

The residual stress of the pulsed electron beam or HIPIB irradiated nanochannel W films was lower than that of the irradiated bulk W. The stress evolution in the nanochannel W films was different from that of the bulk W, where the nanochannel W films had enough free space to hold the thermal expansion and cold shrinkage to slow down the stress accumulation under thermal loads. The work presented here proves that nanochannel W is a promising strategy for the design of PFMs with excellent thermal shock resistance.

## Data Availability

The data that support the findings of this study are available from the corresponding author upon reasonable request.

## Supplementary Materials

The following are available online at https://www.mdpi.com/article/10.3390/nano11102663/s1. Figure S1: Statistical results of the average column size of nanochannel films W-150W-1 (a), W-50W-1 (b), W-150W-10 (c), respectively, Figure S2: The dark-field XTEM image of the W-150W-1 (a) and W-50W-1 (b) film; The bright-field XTEM images of the W-150W-10 film (c), insert show the electron diffraction in region A; (d) is the corresponding GIXRD image, Figure S3: The SEM images of the W-150W-10 film irradiated by HIPIB with the energy density of 1 J/cm^2^ and the pulses of 0 (a), 10 (b), 50 (c) and 100 (d), respectively. The corresponding scale is uniform, Figure S4: The SEM images of the W-150W-10 film and bulk W irradiated by HIPIB; (a–c) are the irradiated W-150W-10 film and (d–f) are the irradiated bulk W with the energy density of 1 J/cm^2^ and the pulses of 10, 50 and 100, respectively. The corresponding scale is uniform, Figure S5: The GIXRD patterns of all samples. The W-150W-1 (a), W-50W-1 (b) films, and bulk W (c) irradiated by pulsed electron beam with the absorbed power density of 0.16, 0.28 and 0.43 GW/m^2^, respectively; the W-150W-10 film (d) and the bulk W (e) irradiated by HIPIB with the energy density of 1 J/cm^2^, respectively. All samples are performed with an angle of incidence α = 5°.
