# Supplementary material for: High Transient-Thermal-Shock Resistant Nanochannel Tungsten Films
Source: Nanomaterials (Basel). 2021 Oct 11;11(10):2663. doi: 10.3390/nano11102663 (PMC8537346; doi:10.3390/nano11102663)
Supplement: Supplementary file 1 [file nanomaterials-11-02663-s001.zip › nanomaterials-1389428-supplementary.pdf]

# High Transient-Thermal-Shock Resistant Nanochannel Tungsten Films

**Tao Cheng [1,†], Wenjing Qin [1,2,†], Youyun Lian [3,*], Xiang Liu [3], Jun Tang [1], Guangxu Cai [1], Shijian Zhang [4], Xiaoyun Le [4,*], Changzhong Jiang [1] and Feng Ren [1,*]**

[1] School of Physics and Technology, Center for Ion Beam Application, Hubei Nuclear Solid Physics Key Laboratory and MOE Key Laboratory of Artificial Micro- and Nano-Structures, Wuhan University, Wuhan 430072, China; juntang@whu.edu.cn (J.T.); jyk@whu.edu.cn (G.C.); czjiang@whu.edu.cn (C.J.)
[2] School of Physics and Electronics, Key Laboratory of Low Dimensional Quantum Structures and Quantum Control, Hunan Normal University, Changsha 410081, China
[3] Fusion Reactor Design & Material Division, Southwestern Institute of Physics, Chendu 610041, China; xliu@swip.ac.cn (X.L.)
[4] School of Physics, Beihang University, Beijing 100191, China; zhangsj@buaa.edu.cn (S.Z.)
† Co-first author: tcheng@whu.edu.cn (T.C.); qwj@hunnu.edu.cn (W.Q.)
* Correspondence: authors email: lianyy@swip.ac.cn (Y.L.); xyle@buaa.edu.cn (X.L.); fren@whu.edu.cn (F.R.)

## Supporting Figures

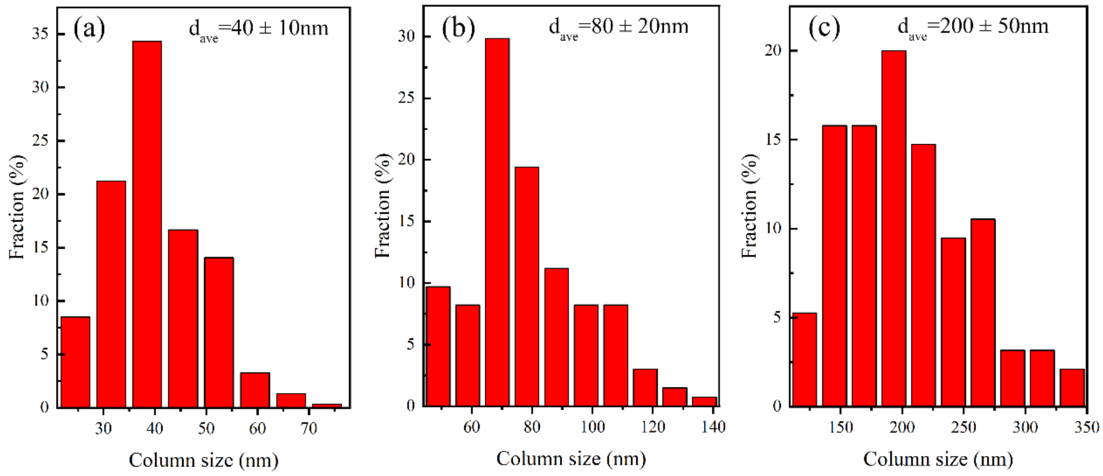


**Figure S1.** Statistical results of the average column size of nanochannel films W-150W-1 (**a**), W-50W-1 (**b**), W-150W-10 (**c**), respectively.

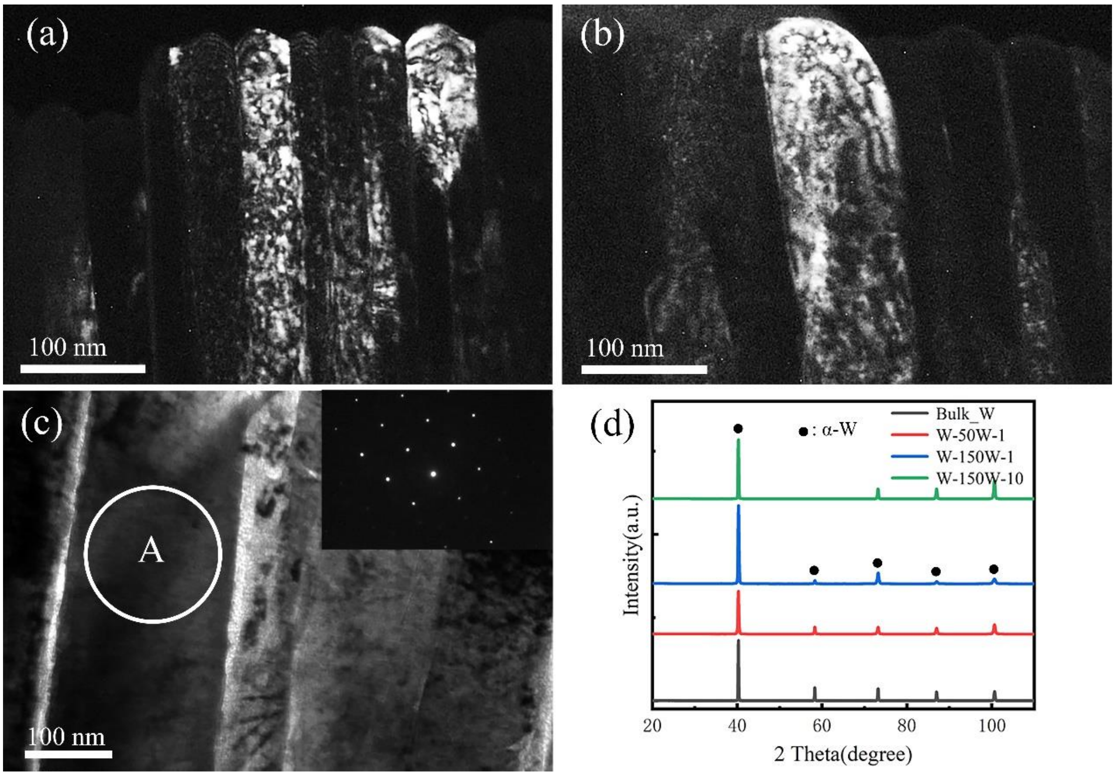


**Figure S2.** The dark-field XTEM image of the W-150W-1 (**a**) and W-50W-1 (**b**) film; The bright-field XTEM images of the W-150W-10 film (**c**), insert show the electron diffraction in region A; (**d**) is the corresponding GIXRD image.

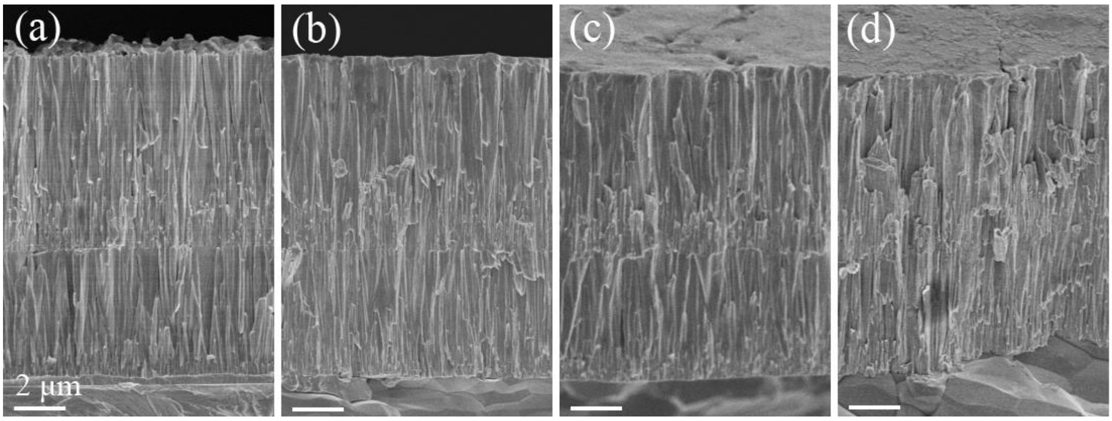


**Figure S3.** The SEM images of the W-150W-10 film irradiated by HIPIB with the energy density of 1 J/cm$^2$ and the pulses of 0 (**a**), 10 (**b**), 50 (**c**) and 100 (**d**), respectively. The corresponding scale is uniform.

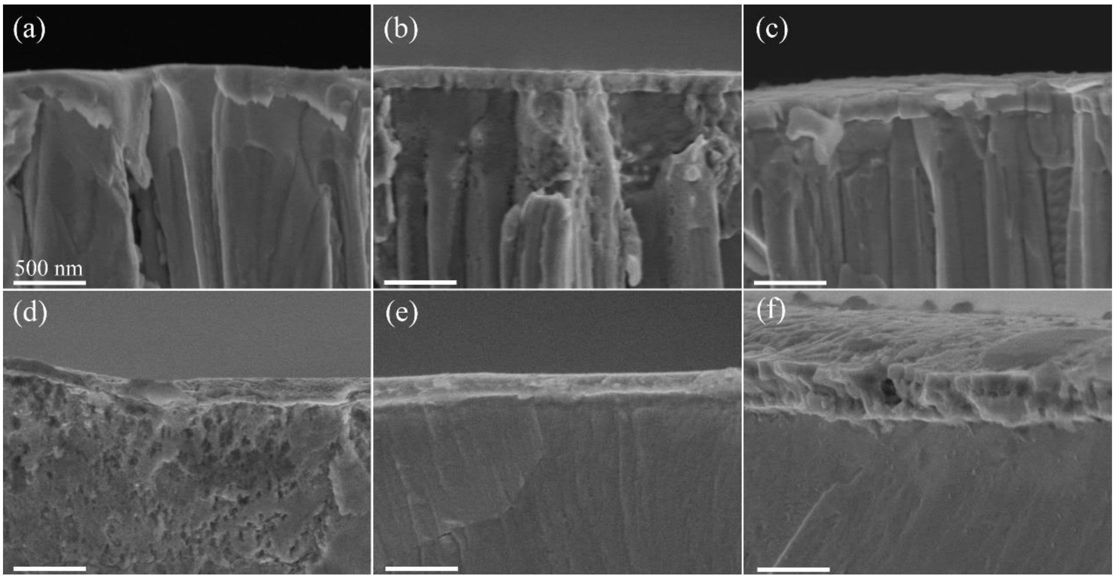


**Figure S4.** The SEM images of the W-150W-10 film and bulk W irradiated by HIPIB; (**a**), (**b**) and (**c**) are the irradiated W-150W-10 film and (**d**), (**e**) and (**f**) are the irradiated bulk W with the energy density of 1 J/cm$^2$ and the pulses of 10, 50 and 100, respectively. The corresponding scale is uniform.

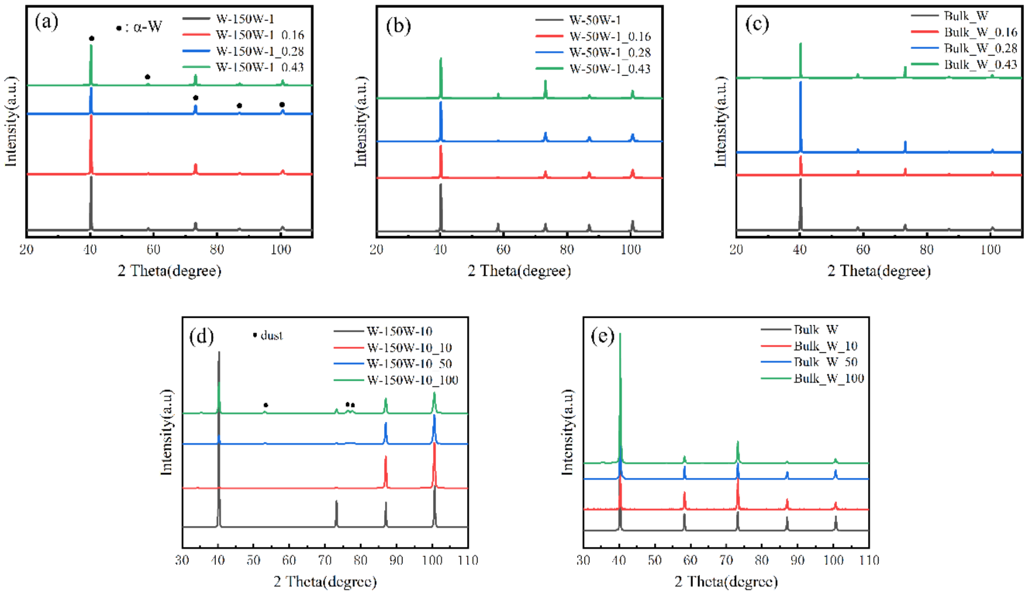


**Figure S5.** The GIXRD patterns of all samples. The W-150W-1 (**a**), W-50W-1 (**b**) films, and bulk W (**c**) irradiated by pulsed electron beam with the absorbed power density of 0.16, 0.28 and 0.43 GW/m$^2$, respectively; the W-150W-10 film (**d**) and the bulk W (e) irradiated by HIPIB with the energy density of 1 J/cm$^2$, respectively. All samples are performed with an angle of incidence $\alpha = 5°$.
